# Adrenaline Dilution in Dental Local Anesthetic Cartridges: A Practical Method Using the Inner Needle of Intravenous Catheter

**DOI:** 10.7759/cureus.76122

**Published:** 2024-12-21

**Authors:** Takutoshi Inoue, Toru Yamamoto

**Affiliations:** 1 Department of Anatomy, Teikyo University School of Medicine, Tokyo, JPN; 2 Division of Dental Anesthesiology, Graduate School of Medicine and Dental Sciences, Niigata University, Niigata, JPN

**Keywords:** adrenaline, cartridge, dilution technique, intravenous catheter, local dental anesthesia

## Abstract

Local anesthesia is a routine medical procedure for dentists. To achieve the desired anesthetic effect of lidocaine and favorable hemostatic effects by adrenaline, the combination of 2% lidocaine + 1:80,000 adrenaline is commonly used, including in dental patients with underlying diseases for whom adrenaline in local anesthetics is problematic due to its vasoconstrictive effects, as the adrenaline concentration in dental local anesthetic cartridges in Japan is commercially set at 1:80,000.

To reduce the effect of adrenaline on the cardiovascular system, adrenaline is sometimes diluted in dental local anesthetic cartridges. We have previously introduced a simple dilution method. However, this method requires the additional purchase of thin metal needles, which may be inconvenient for dentists who do not specialize in dental anesthesia.

Here, we present a more practical dilution method that uses the inner needle of an intravenous catheter (22- or 24-G). Given that intravenous catheters are often kept in dental clinics for sedation or emergency use, we thought that this method would be more versatile. In this method, the inner needle of the intravenous catheter is attached to the syringe and 2% lidocaine without adrenaline is aspirated; then, the half-discarded cartridge is filled with it.

A unique feature of this method is the use of an intravenous catheter, which many dental clinics keep on hand for intravenous sedation or emergencies during dental treatments. To safely administer local anesthesia during dental procedures, we propose a more practical and convenient method of diluting the adrenaline concentration in local anesthetic cartridges.

## Introduction

Dentists use more than 1,500 cartridges of local anesthetics annually, as administering local anesthesia is a routine medical procedure for these professionals [1]. However, when administering local anesthesia to dental patients with underlying diseases, the adrenaline in dental local anesthetics may affect the cardiovascular system owing to its vasoconstricting effects [2]. Therefore, establishing a consensus regarding safe dental treatments using local anesthesia is an important issue [2].

A special glass cartridge (2% lidocaine + 1:80,000 adrenaline) is commonly used for local anesthetics in Japanese dentistry [2]. In Japan, the adrenaline concentrations in local anesthetics used in medical practice are 1:80,000, 1:100,000, and 1:200,000, and the adrenaline concentration can be selected for each patient. However, in dentistry, the adrenaline concentration is set at 1:80,000. Other dental local anesthetics (propitocaine + felypressin, mepivacaine + no vasoconstrictors) can be used for safe dental treatments among patients. However, for patients with underlying conditions where adrenaline may be problematic, 2% lidocaine + 1:80,000 adrenaline may be used to achieve better anesthetic and hemostatic effects.

Therefore, we have previously introduced a simple method for diluting adrenaline in dental local anesthetic cartridges, which is gradually becoming more popular in Japan [2]. This method involves injecting 0.9 mL of 2% lidocaine without adrenaline into a half-discarded cartridge (2% lidocaine + 1:80,000 adrenaline), thereby reducing the adrenaline concentration in the cartridge by half. Diluting adrenaline by half stabilizes vital signs while maintaining anesthetic and hemostatic effects, which is beneficial in dental treatments for hemodynamically unstable patients [2]. However, a potential problem with this dilution method is coring. Coring is the process of inserting a needle into a rubber stopper to scrape off pieces of rubber, which are then mixed with a drug solution [3]. We, therefore, examined the rubber stoppers of 100 used cartridges and found that a 33-G dental injection needle had pierced into the rubber stopper within 1.50 ± 0.08 mm. We concluded that coring could be prevented by slowly piercing the end of the rubber stopper vertically with a 22-G or smaller metal needle [3]. However, for dentists who do not specialize in dental anesthesia, this method requires the purchase of thin metal needles, which may be considered inconvenient. We have devised a more practical dilution method that is convenient for all dentists.

## Technical report

Materials and methods

**Figure 1:**
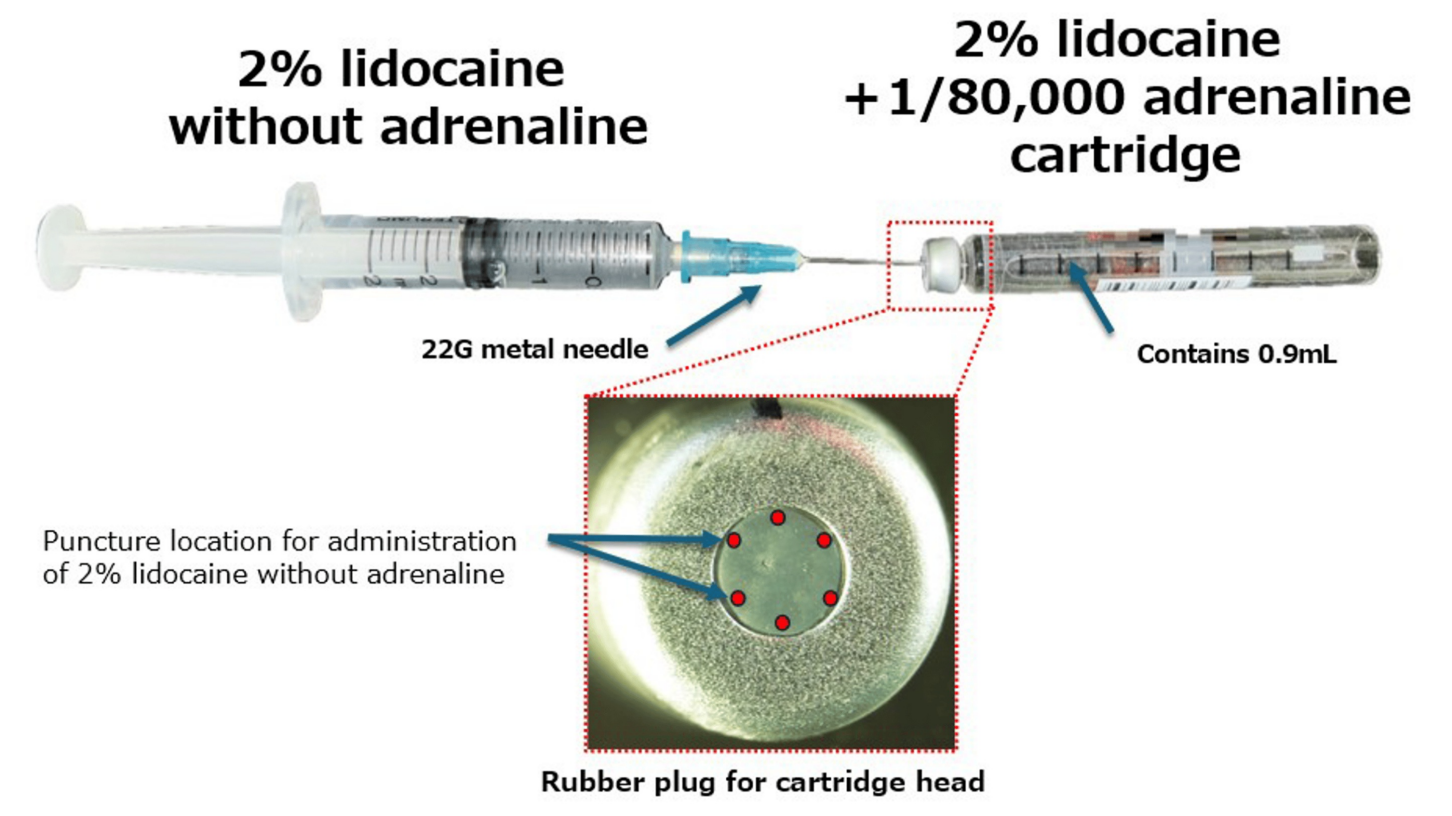
Adrenaline dilution of the cartridge considering coring Insert a ≤ 22-G metal needle into the rubber plug end of a dental local anesthetic cartridge (2% lidocaine + 1:80,000 adrenaline) and inject 0.9 mL of 2% lidocaine without adrenaline into the 0.9 mL cartridge.

For our method, the following items are required: a half-filled dental local anesthetic cartridge (2% lidocaine + 1:80,000 adrenaline), 2% lidocaine without adrenaline, a syringe (3-10 mL), and an intravenous (IV) needle (22- or 24-G). The basic procedure is the same as our previously reported method (Figure 1) [3].

**Figure 2:**
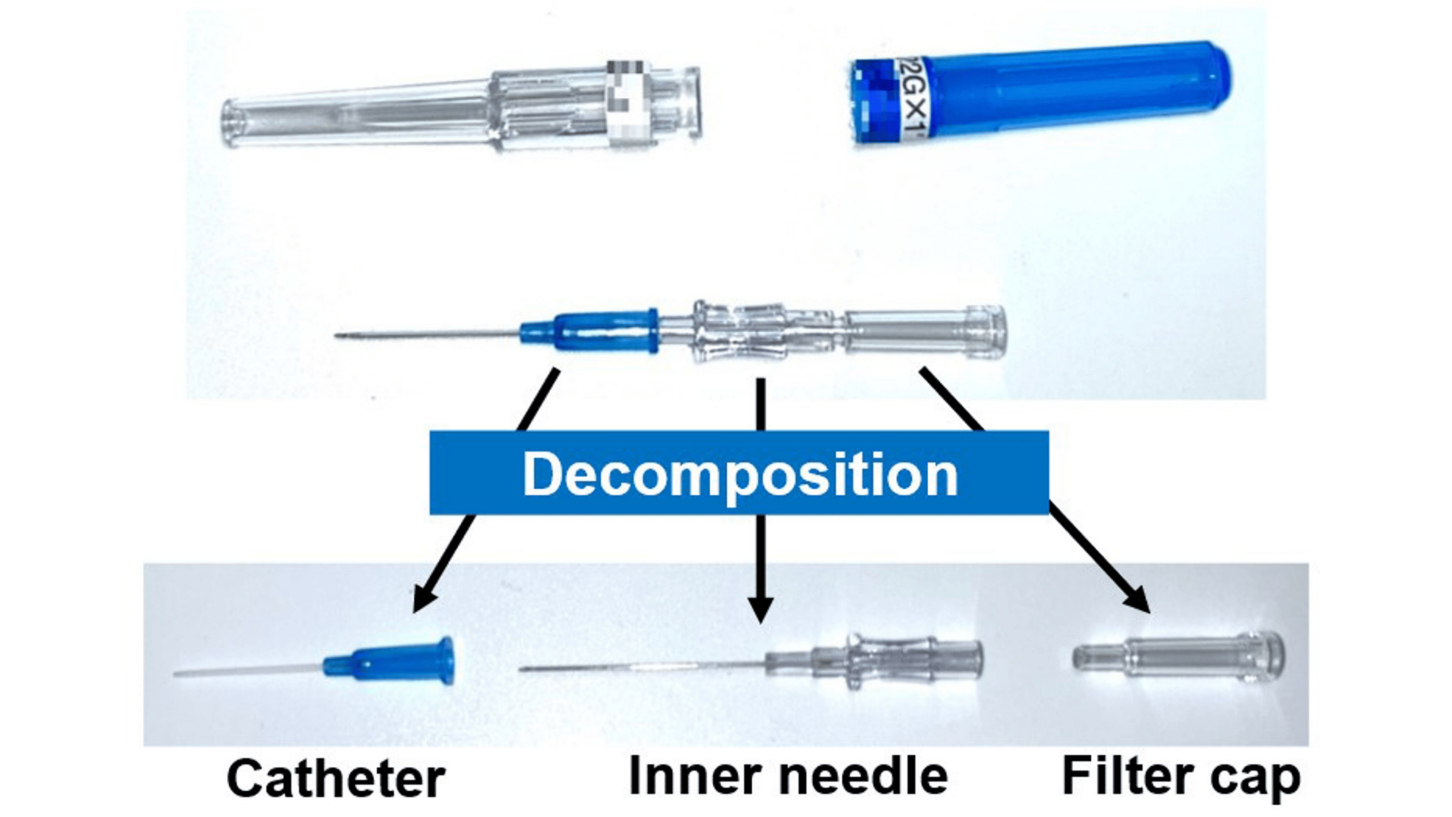
Opening and disassembly of intravenous catheters Open a commonly available intravenous catheter (22-G or 24-G) and disassemble it into a catheter, inner needle, and filter cap. The inner needle of the 22-G catheter is 24-G (outer diameter approximately 0.55 mm).

**Figure 3:**
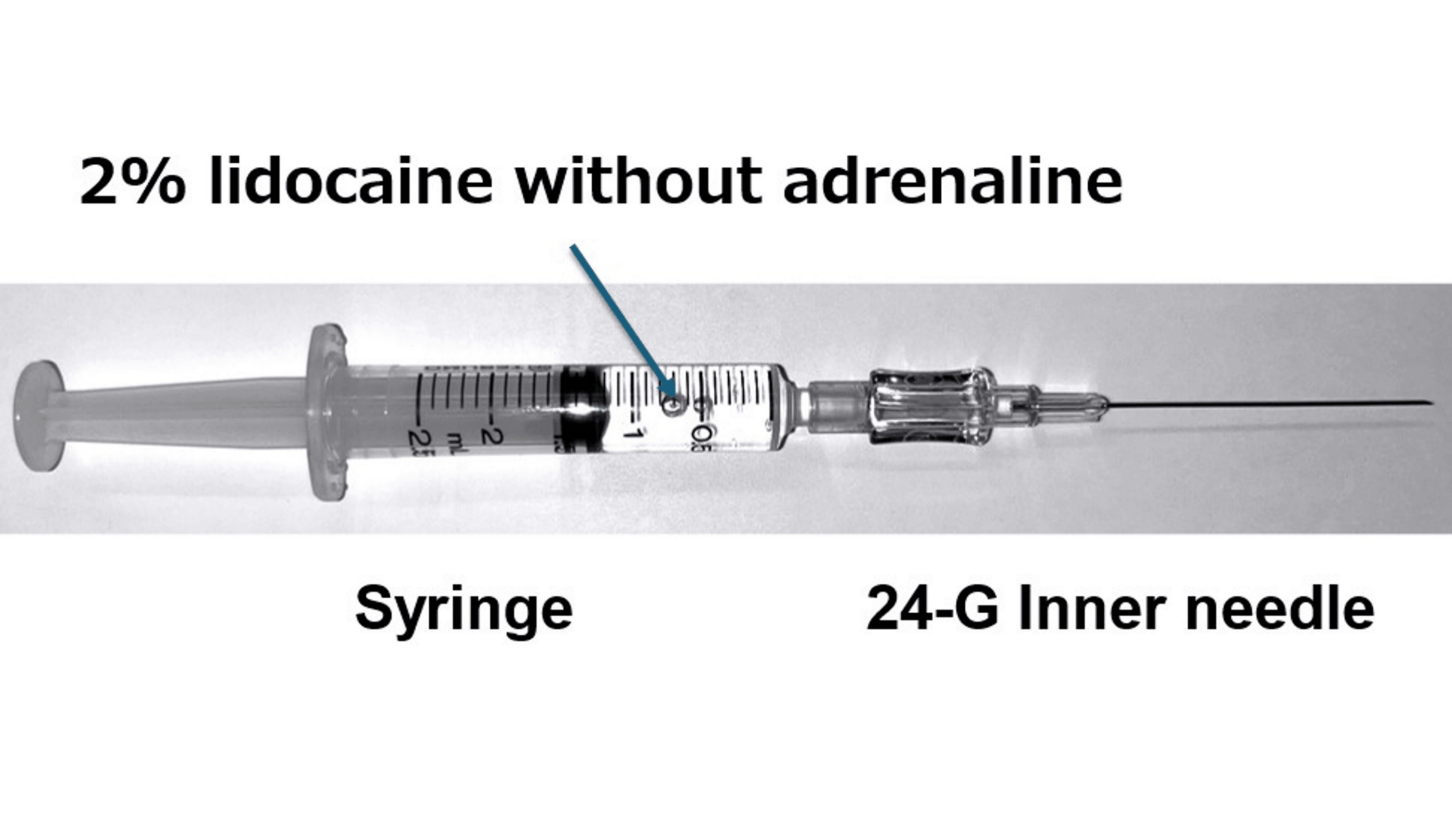
Syringe and inner needle connection Attach a 24-G inner needle to a syringe (3 - 10 mL) and aspirate 2% lidocaine without adrenaline.

**Figure 4:**
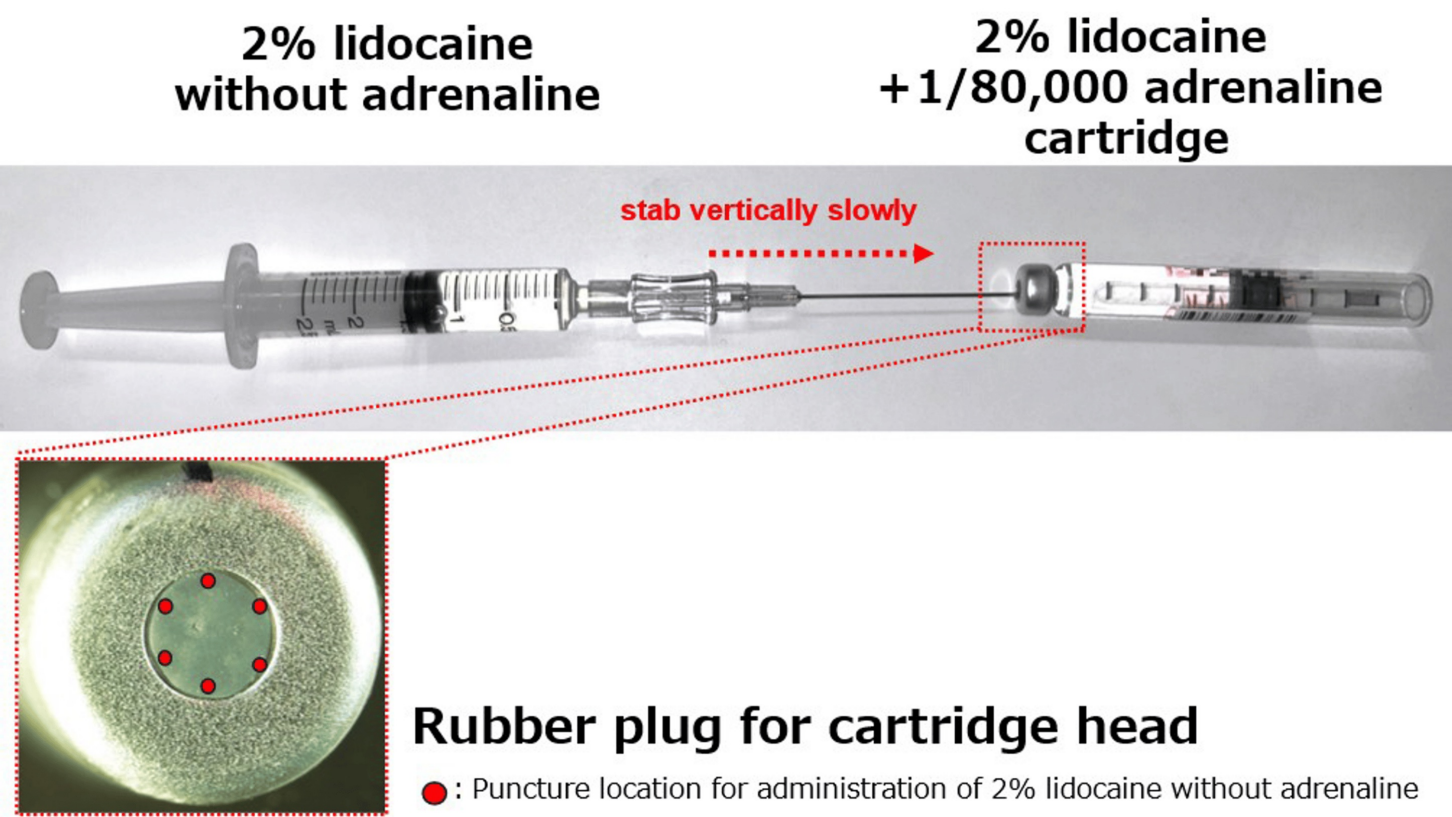
Inner needle puncture position Insert the inner needle into the rubber plug end of the dental local anesthetic cartridge head and inject 2% lidocaine without adrenaline.

In our dilution method, (1) the venous catheter is disassembled into the catheter, inner needle, and filter cap. The inner needle is used for this method (Figure 2). The inner needle of an IV needle (22- or 24-G) meets our reported requirement of 22G or less (outer diameter: ≤0.7mm) [3]. (2) Then, the syringe and inner needle are connected, and 2% lidocaine without adrenaline is aspirated (Figure 3). The needle is long, so careful handling is required. (3) Finally, the adrenaline concentration is diluted by slowly inserting the inner needle vertically into the rubber stopper end of the cartridge head and injecting 0.9 mL of 2% lidocaine without adrenaline (Figure 4) [3]. Be careful not to introduce air bubbles when injecting.

## Discussion

This method was devised with the expectation that general practitioners who are not specialists in dental anesthesia would be able to safely administer local anesthesia, taking into account the adrenaline concentration. In Japan, intravenous sedation (IVS) provided by dental anesthesiologists has become standard and is believed to become increasingly necessary [4]. Additionally, with an increasing elderly population and increased likelihood of medical emergencies occurring during dental treatment, dentists need to be aware of and be confident in managing these situations [5]. Therefore, dental clinics also need to prepare vital sign monitors, automated external defibrillators, sedatives, emergency drugs, IV fluid preparations, IV catheters, and syringes. Given that IV needles and syringes, which should be available on hand in most dental clinics, have a set expiration date, this method is also very useful from the perspective of efficient use of medical resources. Additionally, the investigated IV catheters were 22 G (24-G inner needle, outer diameter: approximately 0.55 mm) and 24 G (27-G inner needle, outer diameter: approximately 0.40 mm), meeting the previously reported requirement of 22 G or less (outer diameter: ≤0.7 mm) [3].

The disadvantage of our method is that unless you have 2% lidocaine without adrenaline available on hand as an emergency medication, you will need to prepare it separately for this procedure. Additionally, because the inner needle of the IV catheter is long, the amount of 2% lidocaine without adrenaline used will slightly increase, and care must be taken to avoid needle-stick injuries. Moreover, IV catheters with needle-stick prevention mechanisms have recently become widely available, and they come in a variety of types, ranging from products with retractable needle tips to products with capped tips. As these products cannot be directly connected to a syringe or have a capped tip and cannot be diluted, our dilution method cannot be used with them.

Although our method has its disadvantages as described above, this technique can be used quickly and easily when administering local anesthesia to dental patients for whom hemodynamic fluctuations must be avoided.

## Conclusions

We introduced a simple and practical method for diluting adrenaline in dental local anesthetic cartridges using the inner needle of an IV catheter. To dilute adrenaline, the inner needle of the IV cannula products is briefly connected to the syringe and slowly inserted vertically toward the end of the rubber stopper at the top of the half-discarded cartridge. This method can be performed by having an IV needle and syringe on hand for IVS or emergencies during dental treatment, and we will present it as a more feasible method.
